# Pharmacological Targets in Chronic Heart Failure with Reduced Ejection Fraction

**DOI:** 10.3390/life12081112

**Published:** 2022-07-24

**Authors:** Maria-Angela Moloce, Irina-Iuliana Costache, Ana Nicolae, Viviana Onofrei Aursulesei

**Affiliations:** 1 Iasi “Saint Spiridon” County Hospital, 700111 Iasi, Romania; angela.moloce@yahoo.com (M.-A.M.); irina.costache@umfiasi.ro (I.-I.C.); onofreiviviana@gmail.com (V.O.A.); 2 Department of Internal Medicine (Cardiology), Iasi “Grigore T. Popa” University of Medicine and Pharmacy, 700111 Iasi, Romania

**Keywords:** heart failure with reduced ejection fraction, novel pharmacological targets, trials

## Abstract

Heart failure management has been repeatedly reviewed over time. This strategy has resulted in improved quality of life, especially in patients with heart failure with reduced ejection fraction (HFrEF). It is for this reason that new mechanisms involved in the development and progression of heart failure, along with specific therapies, have been identified. This review focuses on the most recent guidelines of therapeutic interventions, trials that explore novel therapies, and also new molecules that could improve prognosis of different HFrEF phenotypes.

## 1. Introduction

Optimal medical therapy directed by current heart failure (HF) guidelines is the most important pillar of treatment. Despite clear recommendations, the prognosis for these patients is still marked by high morbidity and mortality rates. Given its significant increase in prevalence worldwide, HF remains an important public health problem. Rapid diagnosis and adequate treatment are essential for these patients. Special attention must be focused on the mechanisms of HF development and targeted therapies [1]. Distinct etiologies, clinical characteristics and comorbidities determine different mechanisms of HF development and progression. Neurohormonal modulation and hemodynamic control proved to be of great benefit, but the analysis of intercellular signaling pathway modulation is believed to have more precise therapeutic potential. Different phenotype-based subgroups related to aging and frequent comorbidities cause myocardial impairment through inflammation and microvascular coronary endothelial dysfunction. Given the heterogeneity of HF patients, several trials have been conducted to evaluate the safety and efficiency of some new molecules. Favorable results will lead to the optimal implementation of novel individualized therapies. Furthermore, present, new, promising and revolutionary research data seem to provide cutting-edge guideline recommendations for HF treatment. Additional targets, such as anatomical and physiological structures (cardiomyocytes and myocardial interstitium, microcirculation), must be considered for novel drug development. Novel therapies addressed to extracellular environment correction, angiogenesis, cellular viability, contractile function or microRNA are also evolving [2,3].

## 2. Materials and Methods

**Figure 1:**
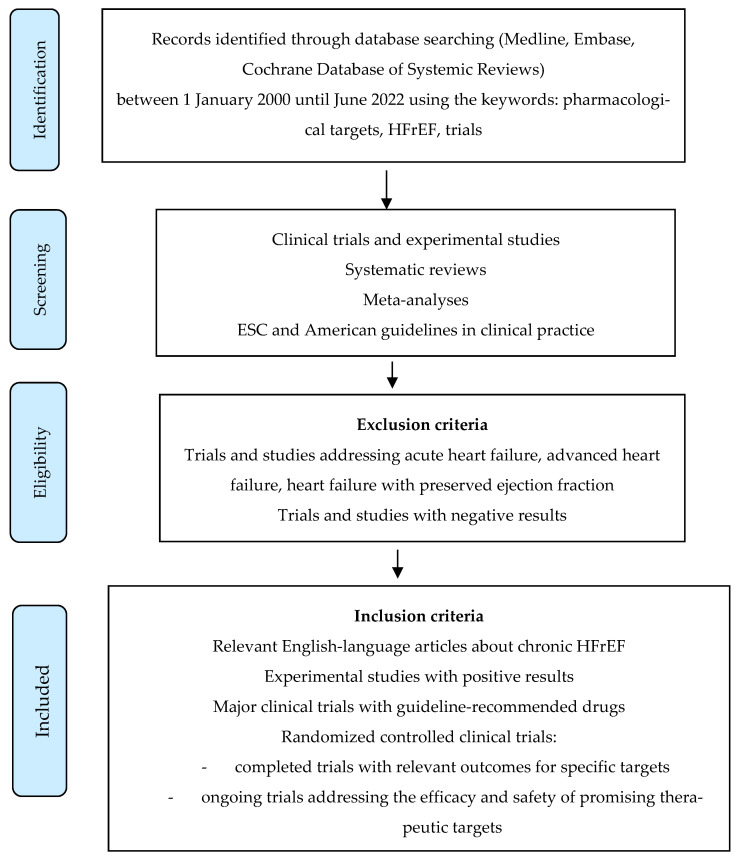
Search strategy flow chart. HFrEF—heart failure with reduced ejection fraction.

Based on most recent data, this review provides an overview of the current guideline-directed medical therapy of HFrEF and novel treatments tested in clinical trials. Our goal was to also present new therapeutic targets based on the progress in our understanding of the molecular and cellular mechanisms leading to HF. A systematic search of MEDLINE, Embase and the Cochrane Database of Systemic Reviews was performed using the following keywords—pharmacological targets, heart failure with reduced ejection fraction, trials. We searched for relevant English-language articles published between 1 January 2000 and June 2022, with a focus on randomized clinical trials, meta-analyses, systematic reviews and clinical practice guidelines. Additional publications were identified during a systematic review of the literature (Figure 1).

## 3. Pathophysiological Mechanisms in HF

The sustained effort to develop a unifying hypothesis to explain the pathophysiology of HFrEF failed to generate a single conceptual model. The development of HFrEF represents the complex interaction between structural and functional biological changes that occur in the heart, autonomic nervous system, kidney, peripheral vascular system and skeletal muscle (Table 1) [4].

HF is a continuously evolving paradigm. In the 1960s to early 1980s, HF was viewed as a hemodynamic model; it was then generally abandoned, except in patients admitted for decompensated HF. Until the late 1960s, congestion and edema were considered a consequence of heart–kidney interaction. In the 1970s, the concept had changed, with HR being viewed as a consequence of pump failure. The concept was based on the Frank–Starling mechanism and the vascular response, known as preload and afterload. From this moment on, the importance of vasodilation as a compensatory mechanism of pump dysfunction was intuited and developed [5]. A more recent hypothesis suggests that HF is a progressive impairment of the left heart, with secondary remodeling due to an index event evolving towards a clinical syndrome consisting of circulatory congestion and loss of cardiac function. This hypothesis suggests the importance of neurohumoral changes represented by sympathetic-adrenergic overactivity, the renin-angiotensin-aldosteron system (RAAS), vasopressin, cytokine activation, increased endothelin levels and natriuretic peptide dysfunction. The role of catecholamines was first suggested by Starling, who described tachycardia and vasoconstriction as mechanisms for increasing cardiac contractility, much later supported by Eugene Braunwald [6]. At the end of the 20th century, RAAS was still in the spotlight and well known for the cascade of long-term adverse cardiovascular effects. Short-term neurohumoral activation is beneficial for increasing pump function, stabilizing blood pressure and maintaining organ perfusion. However, chronic activation disrupts the physiological balance between vasoconstrictor and vasodilator hormones [7]. Therefore, a crucial moment in understanding the mechanisms of HF was the use of the beneficial effects of B-type natriuretic peptides (BNP), with a counter-regulatory effect on RAAS and catecholamines.

Inflammation plays an important role in the pathogenesis of HF and is associated with the up-regulation of proinflammatory cytokines tumor necrosis factor alpha (TNF-α), interleukin 6 (IL-6), interleukin 1α (IL-1α), interleukin 1ß (IL-1ß) and interferon-γ (INF-γ) to the detriment of anti-inflammatory cytokines—interleukin 10 (IL-10) and transforming growth factor beta (TGFß). They initiate the process of apoptosis or necrosis and cause the loss of functional cardiomyocytes. The consequences are LV dysfunction, remodeling and increased collagen turnover of the extracellular matrix, all current targets in HF therapy. Particular attention is paid to TNF-α, considered to be the master regulator of the detrimental inflammatory effects on the heart. This cytokine increases catabolism and is possibly responsible for cardiac cachexia, which may accompany severely symptomatic HF [8]. In turn, inflammation causes microvascular endothelial dysfunction, deficiency in endogenous vasodilator molecules (nitric oxide–NO, prostaglandins) and excess endogenous vasoconstrictor products such as endothelin, a contributor to increased afterload. There is also an enhanced production of reactive oxygen species. Low NO bioavailability causes decreased protein kinase G, cyclic guanosine monophosphate (cGMP) activity and protein kinase C hyperfunction. The consequence is cardiomyocyte hypertrophy and fibrosis, with increased myocardial stiffness and diastolic dysfunction.

A remarkable point of progress in understanding the pathophysiological mechanisms of HF is its relationship with energy metabolism. In HF, there are dramatic changes in energy expenditure and energy supply, with an impact on electrophysiological functions, contractile protein interaction, abnormalities in intracellular calcium modulation and cAMP production. Under normal circumstances, oxidative metabolism in mitochondria provides 95% of cardiac energy, 5% deriving from anaerobic glycolysis. Approximately 70% of cardiac ATP is produced by the oxidation of fatty acids, the remaining 30% coming from the oxidation of glucose and lactate as well as small amounts of ketone bodies and certain amino acids. Most of the energy is consumed to maintain the excitation–contraction coupling, ion flow included. Efficient turnover of metabolic substrates is therefore a prerequisite for normal contractile and energetic function. Deficiencies are closely linked to both cellular oxidative stress and contractile dysfunction. In HF, the energy reserve is depleted and the use of the substrate is deranged, increasing the dependence on glucose metabolism and reducing fatty acid oxidation. The result is a decrease in stored ATP and phosphocreatine (PCr) and a reduction in PCr/ATP ratio, phenomena predictive of adverse events in HF. Given the paradigm of energy depletion in HF, improving cardiac energy metabolism is likely to be an essential target. Hence the undeniable benefit of SGTL2 inhibitors (SGTL2i). They make available an additional source of energy for the heart, namely the circulating ketones resulting from fatty acid mobilization in adipose tissue which are used by the liver for ketogenesis; this is beneficial for increasing functional efficiency of the heart [9].

Finally, intracellular calcium overload is not only caused by decreased myocardial glucose oxidation, but also by impaired function of the sarcoplasmic/endoplasmic reticulum Ca^2+^ -ATPase (SERCA2a), which regulates cardiac myocyte contraction and relaxation by transporting Ca^2+^ from the cytosol into the sarcoplasmic reticulum during diastole. Altered SERCA2a and abnormal handling of Ca^2+^ are associated with HF progression. Contractility and relaxation are both energy consuming processes and depend on ATP hydrolysis [10].

**Figure 2:**
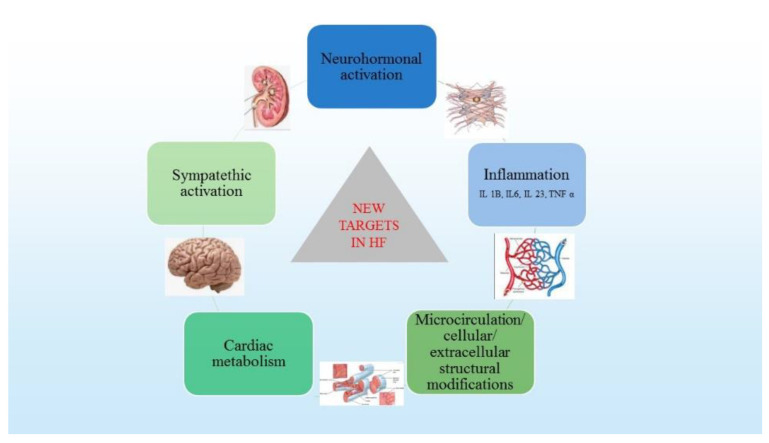
New therapeutic targets in HF (adapted from Corealle et al. [2]).

Overall, all these exhaustive structural changes involve loss of myofilaments, apoptosis, cytoskeleton disorganization, calcium homeostasis disturbance, receptor density changes, signal transduction and collagen synthesis, with devastating functional consequences. The loss of cardiac pump efficiency remains asymptomatic for variable periods of time because of compensatory mechanisms. Once the symptoms occur, these compensatory mechanisms are overwhelmed, determining myocardial damage and inducing disease progression irrespective of neurohormonal status. For all these reasons, the current research focuses on new pathophysiological targets, such as inflammation, cytokine inhibition, cardiac metabolism and cardiomyocytes, without minimizing the role of neurohormonal activation (Figure 2).

## 4. Guideline Recommendations for Therapeutic Targets

During the last three decades, substantial progress has been made in chronic HF management. The main goals of HF treatment are better quality of life, improved symptoms and greater functional capacity in order to prevent disease recurrence and to prolong survival. The newest concept is phenotype-specific HF therapy. The 2021 European guidelines present HF classification according to left ventricular ejection fraction (LVEF) as: HF with reduced LVEF (<40%), HF with preserved LVEF (>50%) and HF with mildly reduced LVEF (40–49%). These three HF classes are characterized by distinct etiologies, comorbidities and demographic features as well as very different responses to treatment. Clinical trials assessing the efficacy of therapeutic agents demonstrated some benefit for HFrEF patients but no improvement in patients with HF with preserved EF. Two more staging systems are presented, one by the American College of Cardiology and American Heart Association (ACC/AHA) and one by the New York Heart Association (NYHA). The ACC/AHA classification is based on structural damage and clinical symptoms of HF, while the NYHA classification follows functional capacity associated with physical activity. Therefore, HF should be considered a multiple stage continuum, with each stage receiving enhanced therapy focused on risk factor modification, structural disease intervention and morbidity and mortality reduction [11,12,13]. The cornerstone treatments for chronic HF are angiotensin-converting enzyme inhibitors (ACE-I) or angiotensin receptor blockers (ARB), beta blockers (BB), mineralocorticoid receptor antagonists (MRA) and diuretics.

### 4.1. Novel Guideline Therapeutic Targets

**Figure 3:**
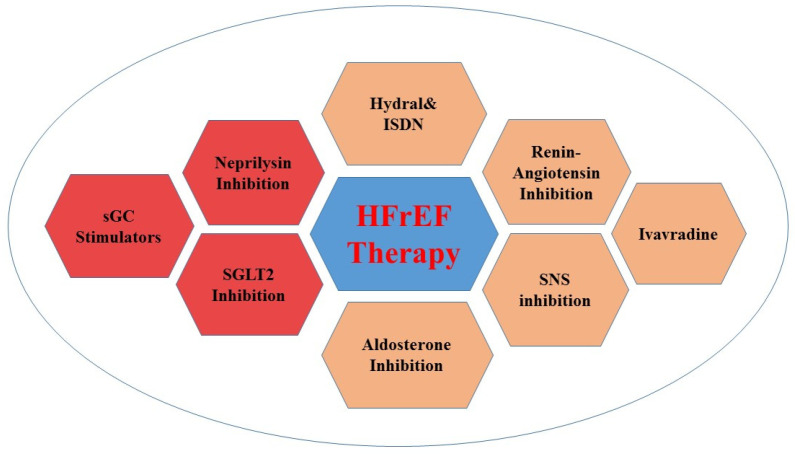
Guideline recommendations for HFrEF therapy.

After nearly two decades without new viable drugs, new classes of drugs for use in HFrEF were approved (Figure 3).

The most popular guideline recommendation is certainly the class of drug referred to as angiotensin receptor–neprilysin inhibitor (ARNI). Their mechanism of action consists of RAAS inhibition and activation of natriuretic peptides. The system includes structurally similar peptides (B and C type) with diuretic, natriuretic and vasodilator effects (mediated by cGMP receptors) as well as antifibrotic and antisympathetic actions. Combining sacubitril, a neprilysin inhibitor, with valsartan has been associated with improvement in HFrEF prognosis. PARADIGM-HF (Prospective comparison of ARNI with ACEI to Determine Impact on Global Mortality and morbidity in Heart Failure), a randomized double-blind trial of 8448 patients with HFrEF (EF < 40%) NYHA class II or III, demonstrated that sacubitril/valsartan was superior to enalapril in reducing the risk of CV death (13.3% vs. 16.5%, *p* < 0.001), HF hospitalizations (*p* < 0.001), prevention of worsening of symptoms (16.7% vs. 14.9%) and quality of life improvement (Table 2) [14].

Based on these results, the 2021 European guidelines recommend ARNI to replace ACE-I in patients with chronic symptomatic HFrEF as a standard therapy (class I, level B), while the 2022 American HF guidelines recommend ARNI as a first-line therapy (class I, level of evidence A) in patients with HFrEF NYHA class II or III so as to reduce mortality and morbidity [11,12]. Sacubtril/valsartan has been approved in symptomatic HF patients. The PIONEER-HF trial (Comparison of Sacubitril/Valsartan versus Enalapril on Effect on NT-proBNP in Patients Stabilized from an Acute Heart Failure Episode) demonstrated that ARNI reduced NT-proBNP levels in patients hospitalized for decompensated acute HF, without an increased rate of adverse events [15]. In the open-label TRANSITION trial (Comparison of Pre- and Post-Discharge Initiation of Sacubitril/Valsartan Therapy in HFrEF Patients After an Acute Decompensation Event), patients with reduced LVEF admitted for HF decompensation were randomized to start ARNI before or after discharge. Safety results were similar in both arms, suggesting that early initiation may simplify management (compared to ACE-I initiation and uptitration and subsequent replacement with ARNI) in the absence of contraindications. ARNI can also be initiated in symptomatic patients with chronic HF and preserved LVEF, but data are limited [16]. Adverse effects, such as hypotension, renal impairment and hyperkalemia, must not be ignored. Pregnancy is a contraindication to ARNI therapy because of its theratogenic potential. Additionally, some hypotheses suggest amiloid cerebral storage and cognitive dysfunction caused by neprilysin inhibition. A PARADIGM-HF trial subanalysis refuted this effect, but long-term studies are needed to confirm it. In this regard, the ongoing PERSPECTIVE trial (Prospective Evaluation of Cognitive Function in Heart Failure: Efficacy and Safety of Entresto compared to Valsartan on Cognitive Function in Patients with Chronic Heart Failure and Preserved Ejection Fraction—NCT02884206) is assessing cognitive decline in 592 patients with chronic heart failure and LVEF above 40% after three years of ARNI treatment compared to valsartan [17].

Another important drug class, newly introduced by guidelines, is sodium-glucose cotransporter 2 inhibitors (SGLT2i), also known as “gliflozins”. They reduce glucose reabsorption by inhibiting sodium–glucose cotransporter 2 found in the proximal nephron tubule. This determines the enhancement of chlorine (NaCl) concentration in the distal tubule and resets the tubuloglomerular feedback mechanism. Consequently, plasma volume contraction is achieved without sympathetic nervous system activation. Empagliflozin, Canagliflozin and Dapagliflozin reduced HF hospitalization rates in diabetic patients at high cardiovascular risk in three main trials: EMPA-REG outcome (Empagliflozin Cardiovascular Outcome Event Trial in Type 2 Diabetes Mellitus Patients), CANVAS program (CANagliflozin cardioVascular Assessment Study—Renal) and DECLARE TIMI 58 trial (Dapagliflozin Effect on CardiovascuLAR Events (Thrombolysis in Myocardial Infarction)) (Table 2). DAPA-HF (Dapagliflozin and Prevention of Adverse-outcomes in Heart Failure) is the first trial to investigate the effects of SGLT2i in HFrEF patients independent of diabetic status. This trial demonstrated a reduction in HF progression of 30% and in cardiovascular death risk of 18%. Moreover, Dapagliflozin reduced all-cause death risk by 17% and improved HF symptoms (hazard ratio, 0.74; 95% confidence interval [CI] 0.65 to 0.85; *p* < 0.001) [18]. Subsequently, EMPEROR-Reduced (Empagliflozin outcome trial in Patients with Chronic Heart Failure with Reduced Ejection Fraction) found that Empagliflozin reduced the combined risk of cardiovascular death and HF hospitalization by 25% in patients with NYHA class II-IV HF, LVEF 40% or less, and elevated natriuretic peptides despite optimal medical therapy (hazard ratio for cardiovascular death or hospitalization for heart failure 0.75; 95% CI 0.65 to 0.86; *p* < 0.001). Exclusion criteria were eGFR < 20 mL/min/1.73 m^2^ in EMPEROR-Reduced or <30 mL/min/1.73 m^2^ in DAPA-HF, type 1 diabetes, or systolic blood pressure < 95 to 100 mmHg [19]. Therefore, Dapagliflozin and Empagliflozin are recommended for all patients with HFrEF, regardless of the presence of diabetes (class I, level of evidence A), according to the European and American guidelines for HF [11,12]. The paradigm shift in HF therapy also includes adapting the medication to patient phenotype. From this point of view, Dapagliflozin works in most subgroups of HFrEF patients, namely patients with atrial fibrillation, systolic blood pressure > 95–100 mmHg or hypertension, high/low heart rates, hyperkalemia, chronic kidney disease and type 2 diabetes, respectively. Dapagliflozin and Empagliflozin have been shown to be effective and safe in improving cardiovascular and renal targets in patients with eGFR > 25 mL/min/1.73 m^2^ and 20 mL/min/1.73 m^2^, respectively. However, there is evidence of benefits in relation to the use of Dapagliflozin in patients with eGFR < 20 mL/min/1.73 m^2^ [20]. It is worth mentioning the modest and reversible decrease in eGFR in the first days after the initiation of SGLT2i, which does not require discontinuing medication because of the documented long-term beneficial effect on renal function. On the other hand, SGTL2i is not the perfect drug. Increased attention should be paid to maintaining euglycemic status, the risk of ketoacidosis, genital and soft tissue infections and, if necessary, adjusting diuretic treatment to prevent severe volume depletion [19]. In the SOLOIST-WHF (Effect of Sotagliflozin on Cardiovascular Events in Patients with Type 2 Diabetes and Worsening Heart Failure), a multicenter double-blind trial was conducted in 1222 patients with diabetes and recent HF hospitalization who were enrolled before discharge or within 3 days after discharge. The median follow-up time was 9.0 months. Sotagliflozin, a dual inhibitor of sodium–glucose cotransporters 1 and 2, reduced the combined endpoint of cardiovascular death, HF hospitalization or urgent HF visits by 33% (hazard ratio, 0.67; 95% CI 0.52 to 0.85; *p* < 0.001) but has not been approved by the US Food and Drug Administration (FDA). An essential aspect of HF management is the firm recommendation of both guidelines to associate ACE-I/ARNI, SGLT2i, beta blockers and aldosterone antagonists as early as possible, and to not administer them separately in a sequential manner [21].

Vericiguat, an oral soluble guanylate cyclase (GCs) stimulator that raises the production of cyclic guanylate monophosphate (cGMP), is also a new drug introduced in current guidelines. Phase 2 studies showed that Vericiguat is well tolerated by patients with HFrEF. The VICTORIA trial (Vericiguat Global Study in Subjects with Heart Failure with Reduced Ejection Fraction), a phase 3 randomized double-blind study, has evaluated the effects of Vericiguat on 4500 patients with chronic HFrEF and demonstrated a reduction in cardiovascular death (16.4% in Vericiguat group vs. 17.5% in placebo group; hazard ratio, 0.93; 95% CI, 0.81 to 1.06) and HF hospitalizations (37.9% in Vericiguat group vs. 40.9% in placebo group; hazard ratio, 0.90; 95% CI, 0.83 to 0.98; *p* = 0.02) (Table 2). Therefore, this drug is also recommended in the current HF guidelines (class II, level of evidence B) [22]. It has been demonstrated that deficiency in sGC-derived cyclic guanosine monophosphate (cGMP) induced by low NO bioavailability leads to myocardial dysfunction and endothelial dysfunction in coronary microcirculation. Nitric oxide activates guanylyl cyclases (GCs), followed by a rise in cGMP level in the vascular and nonvascular tissues, such as myocardium and kidney tissues. In HF, NO production is low, resulting in high arteriolar, pulmonary venous and systemic tone and leading to increased cardiac preload and afterload. In the myocardium, NO modulates the calcium channel activity, SERCA pump, sarcoplasmic reticulum and ryanodine receptor and has complex effects on mitochondrial metabolism. Different NO-synthase isoforms are also involved in the ventricular remodeling process. An increase in oxygen free radicals and a decrease in NO production are detected in HF, determining disease progression and reduction in beneficial vasodilator effects [10]. Thus, restoration of adequate nitric oxide (NO)-sGC-cGMP signaling is an important therapeutic target.

Another mechanism of HF progression is fast heart rate (HR). This reflects the imbalance between sympathetic hyperstimulation and parasympathetic inhibition, both components of neurohormonal activation. New studies demonstrate the contribution of this mechanism to high cardiovascular death and HF hospitalization rates, thus making it a new potential therapeutic target. Multiple drugs, such as BB, Digoxin, Amiodarone and Ivabradine, can modulate HR. Ivabradine is an If channel inhibitor in the sinoatrial node which controls spontaneous diastolic depolarization. It is recommended in patients with HFrEF and a LVEF less than 35% in sinus rhythm with HR over 70 beats per minute and who remain symptomatic despite maximum tolerated doses of BB therapy. The SHIFT trial (Systolic Heart Failure Treatment with the If Inhibitor Ivabradine) demonstrated a reduction in HF hospitalizations and death rates of 18% (HR 0.82; 95% CI, 0.75–0.90, *p* < 0.0001) (Table 2) [23]. Hence, Ivabradine is recommended by both the European and American HF guidelines (class I, level of evidence A) [11,12].

Aldosterone plays an important role in the pathophysiology of HF by inflammation, vascular rigidity, collagen synthesis and myocardial necrosis. At the same time, chronically high aldosterone levels are associated with coronary and renovascular remodeling, endothelial and baroreceptor dysfunction, and myocardial hypertrophy. Adding an MRA drug to standard HF therapy improves survival and reduces mortality in symptomatic chronic HF patients and also in subgroups with systolic dysfunction following myocardial infarction. Spironolactone was first studied in the RALES trial (Randomized Aldactone Evaluation Study). A blockade of aldosterone receptors by spironolactone, in addition to standard therapy, substantially reduced the risk of both morbidity and death among patients with severe HF (relative risk of death 0.70; 95% CI, 0.60 to 0.82; *p* < 0.001). Because of its antiandrogenic adverse effects (gynecomastia and breast sensitivity in males), eplerenone, a selective MRA with fewer endocrine side effects, was developed. It was investigated in EPHESUS (Eplerenone Post–Acute Myocardial Infarction Heart Failure Efficacy and Survival Study), where the rate of the primary endpoints—death from cardiovascular causes or hospitalization for cardiovascular events—was reduced (relative risk 0.87; 95% CI, 0.79 to 0.95; *p* = 0.002), as was the secondary endpoint—death from any cause or any hospitalization (relative risk, 0.92; 95% CI, 0.86 to 0.98; *p* = 0.02) (Table 2). It was also studied in the EMPHASIS-HF (Eplerenone in Mild Patients Hospitalization and Survival Study in Heart Failure) trial which demonstrated similar beneficial effects and less adverse effects compared to spironolactone (hazard ratio, 0.63; 95% CI, 0.54 to 0.74; *p* < 0.001) (Table 2). Based on these data, MRA treatment is recommended in all HF patients who remain symptomatic, despite ACE-I, ARB or ARNI treatment with a BB, to reduce HF hospitalizations and death (class I, level of evidence A) [11,12].

### 4.2. New Approaches for Studying the Additional Benefits of Some Previous Therapeutic Targets

Digoxin is the oldest, but also the most controversial, drug prescribed in HF therapy (class II, level of evidence B) according to the European HF guidelines. In the DIG study, it did not reduce mortality compared to placebo, though it did reduce HF hospitalizations (26.8 percent vs. 34.7 percent; risk ratio, 0.72; 95% CI, 0.66 to 0.79; *p* < 0.001). A retrospective analysis suggested that patients with a digoxin blood level between 0.5 and 0.9 mg/mL have some benefits [24,25]. An ongoing prospective placebo-controlled trial (DECISION trial—NCT03783429) is currently testing if lower digoxin doses guided by blood levels will reduce HF hospitalizations and cardiovascular death rate in approximately 1000 patients (https://clinicaltrials.gov/ct2/show/NCT03783429; accessed on 3 July 2022). The results will be published in 2025 (Table 3).

Furthermore, a combination of hydralazine and isosorbide dinitrate may be considered (class II, level of evidence B) to reduce mortality in symptomatic HFrEF patients who do not tolerate ACE-I, ARNI or ARB or who are contraindicated [11,12].

Congestion, an important cause of the signs and symptoms of HF, causes atrial and ventricular remodeling, arrhythmias and renal impairment and is a predictor of poor prognosis. Congestion treatment is an essential part of HF management. However, its diagnosis remains a challenge. In some patients without clinical evidence of congestion, subclinical signs have been demonstrated using pleural and cardiac ultrasound, either in the interstitial space (lung B lines) or in the intravascular space (inferior vena cava distention). Loop diuretics are the mainstay of decongestion therapy. Although the most widely used loop diuretic is Furosemide, Bumetanide and Torsemide are better absorbed and released in the renal tubule. A meta-analysis of some small randomized and observational studies suggested the possible superiority of Torsemide compared to Furosemide; however, there are no randomized studies to verify this [26].

TRANSFORM-HF (ToRsemide compArisoN With furoSemide FOR Management of Heart Failure), an ongoing multicenter study, will randomize 6000 patients with decompensated HF before discharge to compare the effectiveness of Torsemide versus Furosemide and its effects on mortality and morbidity (Table 3) [27]. Resistant congestion can be managed by combining different classes of diuretics, although the efficacy of this strategy has not been tested in clinical trials.

Most of the sodium is reabsorbed in the proximal tubule of the nephron. Acetazolamide, a carbonic anhydrase inhibitor, decreases proximal tubular sodium reabsorption, therefore enhancing the effect of loop diuretics. ADVOR (Acetazolamide in Decompensated Heart Failure With Volume OveRload), a randomized double-blind placebo-controlled study which will enroll 500 HF patients, is planning to test the efficiency of this association (Table 3) [28]. Tolvaptan is an oral vasopressin type 2 receptor antagonist that enhances water excretion through the collecting tubules. Urine volume is high, but without enhanced electrolyte excretion. The EVEREST study (The Efficacy of Vasopressin Antagonism in Heart Failure Outcome Study With Tolvaptan) compared the safety and efficacy of tolvaptan versus placebo in the treatment of patients with worsening congestive HF and showed relief of dyspnea. The composite endpoint of cardiovascular death or hospitalization for HF occurred in 871 tolvaptan group patients (42%) and 829 placebo group patients (40.2%) (hazard ratio, 1.04; 95% CI, 0.95–1.14; *p* = 0.55) (Table 3). Likewise, in the QUEST study (Efficacy and Safety of Tolvaptan in Heart Failure Patients with Volume Overload Despite the Standard Treatment with Conventional Diuretics), tolvaptan was associated with weight loss, relief of HF symptoms and signs and increased diuresis. Over a 3-year period in the tolvaptan group, the increase in total kidney volume was 2.8% per year (95% CI, 2.5 to 3.1) versus 5.5% per year in the placebo group (95% CI, 5.1 to 6.0; *p* < 0.001). The composite endpoint favored tolvaptan over placebo (44 vs. 50 events per 100 person-years, *p* = 0.01), with lower rates of worsening kidney function (2 vs. 5 events per 100 person-years, *p* < 0.001) and kidney pain (5 vs. 7 events per 100 person-years, *p* = 0.007). Nevertheless, some patients developed hypernatremia at high tolvaptan doses (Table 3) [29].

## 5. Evolving Therapeutic Targets

**Figure 4:**
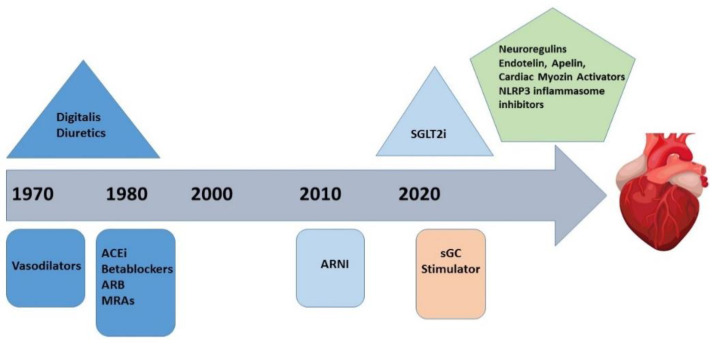
Progression of therapeutic targets over time.

Despite the encouraging results obtained with the new molecules already introduced in HF guidelines, the battle with this disease is not fully won. The discovery of some new mechanisms involved in HF development offers attractive perspectives for the research of some new molecules with additional benefits. On the other side, most hospitalizations are related to worsening of symptoms and signs of chronic HF (acutely decompensated HF), a highly heterogeneous pathophysiologic condition. There is a large amount of information regarding the new strategies in chronic HF. Interesting therapeutic molecules targeting the causative mechanisms of HR decompensation are also intensively studied (Figure 4).

Finerenone, a novel non-steroidal MRA, combines spironolactone efficiency with eplerenone selectivity resulting in a lower level of hyperkalemia and a more significant decrease in natriuretic peptide levels. The ARTS-HF trial (Miner Alocorticoid Receptor antagonist Tolerability Study-Heart Failure) was a randomized, double-blind, phase 2b, multicenter study enrolling 1066 patients who received oral, once-daily finerenone or eplerenone. The study demonstrated a decrease of >30% in plasma N-terminal pro-B-type natriuretic peptide over 90 days in 37.2% of patients as a primary endpoint. The proportion of patients was similar between the subgroups. The composite endpoint included cardiovascular hospitalizations, acute worsening HF or 90-day all-cause mortality. The composite endpoint was statistically significant only in the 10 to 20 mg group (hazard ratio, 0.56; 95% CI, 0.35; 0.90; *p* = 0.02); however, further research is needed to confirm these findings (Table 3) [33].

Achieving optimal heart pump performance requires the use of significant amounts of ATP and therefore relies on such substrates as carbohydrates and fatty acids for energy requirements. In HF, insufficient ATP is generated by defects in glycolysis, oxidation of glucose and fatty acids and oxidative phosphorylation. Perhexilline is a metabolic modulator whose main action appears to be the inhibition of fatty acid oxidation by inhibiting carnitine palmitoyltransferases 1 and 2 (CPT-1/2). These are specific proteins that carry fatty acids from the cytoplasm into the mitochondria. After CPT-1/2 inhibition, beta-oxidation is reduced, shifting the metabolism to glycolysis and increasing mechanical efficiency, thus improving the efficiency of ATP generation. In a phase 2 randomized, double-blinded, placebo-controlled study using direct measures of energy metabolism in the left ventricle, cardiac magnetic resonance spectroscopy showed a clear biological effect on human cardiac metabolism but no changes in noninvasively assessed contractile function or invasive assessment of myocardial substrate utilization after 1 month of therapy (Table 3) [38]. Despite these results, the study is a strong model for a new phenotyping approach in HF patients.

Cardiotrophin 1 cytokine (CT1) is a new molecule that belongs to the IL-6 family. Initially discovered as a factor inducing cardiomyocyte hypertrophy, it has a variety of different effects, including the ability to stimulate the survival of cardiac and neuronal cells. Interestingly, although activation of the p42/p44 MAP kinase pathway is necessary for promoting the survival effects of CT1 in the cardiac cells, it is not necessary for its hypertrophic effect that is probably secondary to the activation of the Jak/STAT-3 pathway. Thus, CT-1 may be used as a new cardioprotective agent, especially if its hypertrophic effect can be specifically inhibited. In addition, CT1 has been shown to enhance the production of heat shock proteins hsp70 and hsp90, which protect cardiomyocytes from thermic and ischemic stress. Moreover, it seems that CT1 reduces the tumor necrosis factor, with beneficial effects in myocardial infarction and ischemic HF. In HF, CT1 promotes beneficial cardiac remodeling, reduces pathological cardiac structural and ischemic changes and also regulates obesity secondary to increased food intake and insulin resistance [39,40].

Interleukin 1 (IL-1) beta is also known to depress cardiac function. IL-1 inhibition has beneficial effects in HF because the acute inflammatory response is suppressed, thrombotic cardiovascular events are prevented, and cardiac function and patient quality of life are improved [41]. The extension phase of the CANTOS study [Cardiovascular Risk Reduction Study (Reduction in Recurrent Major CV Disease Events)], a randomized double-blind trial, suggests Il-1 as a potential therapeutic target in HF. Canakinumab, an IL-1β antibody, at a dose of 150 mg every 3 months significantly reduced the composite endpoint of HF hospitalization or HF–related mortality in 10,061 patients with a history of acute myocardial infarction (hazard ratio vs. placebo, 0.83; 95% CI, 0.73 to 0.95; *p* = 0.005) (Table 3) [35]. In the REDHART trial (the REcently Decompensated Heart failure Anakinra Response Trial), Anakinra, a recombinant human Il-1 receptor antagonist, improved peak aerobic exercise capacity after 12 weeks of treatment in patients with LVEF < 50% (from 14.5 mL/kg/minute to 16.1 mL/kg/minute; *p* = 0.009) (Table 3) [36]. A currently underway single center, randomized, placebo-controlled, double-blind, phase II, randomized clinical trial is evaluating the 24-week effect of anakinra on cardiorespiratory capacity in patients with recent hospitalization for acute HFrEF decompensation [30].

Dapansutrile (OLT1177) is a selective oral NLRP3 inflammasome inhibitor (intracellular sensor that detects endogenous danger signals, environmental irritants) aimed at inhibiting IL-1β and IL-18 activity. Recently, a randomized, double-blind, phase IB study on dapansutrile in 30 patients with stable HFrEF, NYHA class II or III reported that it is safe and well-tolerated over the 14-day treatment regardless of the administered doses. For example, in the group receiving 2000 mg of dapansutrile, LVEF and exercise time were improved (from 31.5% to 36.5%, *p* = 0.039 and from 570 to 616 s, *p* = 0.039, respectively) [42]. Endothelin antagonists seem to be a promising therapeutic target for HF, considering their role in pathological fibrosis, hypertrophy, arterial hypertension and overregulation. Despite encouraging preclinical data, some trials did not report significant benefits, while others reported adverse effects. This is probably secondary to the competitive effects in different cell types or receptor subtype selectivity [43].

Neuregulin-1 proteins are important in the development and function of cardiomyocytes. Some small phase 2 studies using recombinant human neuregulin-1 reported improved hemodynamics and reverse remodeling in HFrEF patients [44]. A phase 3 study (NCT03388593—Survival Study of the Recombinant Human Neuregulin-1β in Subjects With Chronic Heart Failure) is ongoing and seeks to assess the efficiency and safety of daily intravenous neuregulin-1 perfusion, followed by weekly bolus in HFrEF, on all-cause mortality (https://clinicaltrials.gov/ct2/show/NCT03388593; accessed on 3 July 2022) (Table 3).

Patiromer and sodium zirconium cyclosilicate are new oral treatments that bind potassium into the gastrointestinal tract and rapidly normalize serum potassium levels. Their use is still discussed, and they are not a guideline recommendation. Patiromer and sodium zirconium cyclosilicate could be useful in association with ACE-I, ARB or ARNI for reaching the maximum recommended doses, considering that these drugs should not be initiated if serum potassium level is over 5 mmol/L. Doses should be reduced, or therapy interrupted, if potassium serum level reaches 5.5 mmol/L. Hyperkalemia is a frequent finding in HF-related diabetes mellitus, chronic kidney disease or elderly patients. BIOSTAT-CHF (BIOlogy Study to TAilored Treatment in Chronic Heart Failure), an international, multicenter, prospective, observational study was specifically designed to assess the effects of ACE-I/ARB uptitration and its association with outcome. The study concluded that higher potassium levels are an independent predictor of enduring lower dosages, without attenuating the beneficial effects of uptitration [45]. The DIAMOND (Patiromer for the Management of Hyperkalemia in Subjects Receiving RAASi Medications for the Treatment of Heart Failure) trial, a prospective phase 3b multicenter, double-blind, placebo-controlled, randomized study, enrolled 878 eligible patients with HFrEF. The follow-up time was 13.0 weeks. Subjects with hyperkalemia (serum potassium level over 5.5 mmol/L) were randomized to patiromer (*n* = 439) versus control group (*n* = 439). The trial included a run-in phase during which the patients received patiromer and optimized doses of RAAS inhibitors and a randomized withdrawal blinded treatment phase. The primary endpoint, adjusted mean change in serum potassium level, was 0.03 mEq/L in the patiromer group versus 0.13 mEq/L in the control group (*p* < 0.001). Patiromer seems to be a beneficial strategy for managing patients at risk of hyperkalemia. Its administration also permits 85% of patients to receive appropriate doses of RAAS inhibitors (Table 3) [32].

Iron deficiency is a common comorbidity in HF patients and is associated with reduced quality of life and functional capacity, higher rates of hospitalization and mortality, regardless of the presence of anemia. Intravenous iron supplementation is a promising therapeutic target in HF patients given its essential role in mitochondrial aerobic respiration and cellular immune response, including cardiomyocytes. The European guidelines recommend that HFrEF patients should be tested for anemia and iron deficiency using serum ferritin and transferrin saturations. The European guidelines also recommend intravenous ferric carboxymaltose administration in symptomatic HF patients with documented iron deficiency to improve symptoms and quality of life (class IIa, Level of Evidence A recommendation). American guidelines support intravenous iron treatment as a class IIb, level of evidence B recommendation [11,12]. The FAIR-HF (Ferric Carboxymaltose Assessment in Patients With Iron Deficiency and Chronic Heart Failure) trial included 459 patients with chronic HF who received weekly intravenous ferric carboxymaltose until iron repletion. The results sustained significant improvements in NYHA functional class at week 24 (odds ratio for improvement by one class, 2.40; 95% CI, 1.55 to 3.71; *p* < 0.001), in distance on the 6-min walk test and in quality of life (evaluated by the European Quality of Life–5 Dimensions Visual Analog Scale and Kansas City Cardiomyopathy questionnaire) at weeks 4, 12 and 24 (*p* < 0.001 for all comparisons) [31] (Table 3). CONFIRM-HF (Ferric CarboxymaltOse evaluatioN on perFormance in patients with IRon deficiency in coMbination with chronic Heart Failure) was a double-blind, multi-center, prospective, randomized, placebo-controlled trial which enrolled 304 ambulatory patients with symptomatic HFrEF, iron deficiency (defined as ferritin < 100  ng/mL, or ferritin 100–300  ng/mL if transferrin saturation < 20%) and haemoglobin < 15  g/dL. The trial provided evidence that treatment with intravenous feric carboximaltose over one year improved exercise capacity, symptoms and quality of life, and is thus associated with a reduced risk of hospitalizations due to worsening HF (hazard ratio: 0.39, *p* *=* 0.009) [37] (Table 3). A more recent meta-analysis included data from four randomized trials using the same intravenous iron preparation (ferric carboxymaltose). FAIR-HF and CONFIRM-HF contributed approximately 90% of the total number of subjects included. This meta-analysis concluded that intravenous ferric carboxymaltose in patients with iron deficiency may decrease recurrent hospitalizations in HFrEF [46]. Three large randomized trials studying the benefits of intravenous iron on mortality and hospitalizations rates in chronic HFrEF are still active (FAIR-HF2: https://clinicaltrials.gov/ct2/show/NCT03036462; HEART-FID: https://clinicaltrials.gov/ct2/show/NCT03037931 or currently recruiting IRONMAN: https://clinicaltrials.gov/ct2/show/NCT02642562; accessed on 16 July 2022) (Table 3).

HF may be associated with high plasma copper concentrations, but also with myocardial copper depletion. Experimental models suggest that copper chelation is beneficial for HF patients. Small doses of Trientine, an alternative copper chelator, could facilitate redistribution of copper into the tissues. The ongoing TRACER-HF trial (NCT0387518—Study to Evaluate Effects of INL1 in Patients With Heart Failure and Reduced Ejection Fraction), a multicenter, randomized, double-blind, placebo-controlled, dose–response study, is evaluating the efficacy and safety of three PO INL1 (a copper-binding agent) doses in 200 patients with chronic, stable HFrEF compared to placebo. The primary outcome measure is the decrease in serum NT-proBNP level from baseline to 12 weeks. The trial will also assess the effect on echocardiographic parameters and functional status [https://clinicaltrials.gov/ct2/show/NCT03875183; accessed on 3 July 2022] (Table 3) [47].

Coenzyme Q10 is an essential component of the mitochondrial electron transport chain and has an important role in the metabolic process. Lower selenium and coenzyme Q10 concentrations have been associated with adverse HF progression. Some randomized trials and meta-analyses sustain the efficacy of coenzyme Q10 in improving functional status and echocardiographic parameters in HFrEF, though its prognostic role is still debated [48,49,50].

Apelin was discovered in 1993 as an orphan G protein-coupled receptor. Its protein structure is highly similar to that of angiotensin II receptor type 1 (AT1), although no binding to the receptor was observed with angiotensin II. Apelin attenuates ventricular hypertrophy and stimulates contractility in failing cardiac muscle by increasing the availability of intracellular calcium rather than enhancing myofilament calcium sensitivity. Therefore, it is a promising therapeutic agent in HF. Many apelin receptor agonists have been used both in vitro and in vivo. The 2021 study by Gargalovic et al. presents a new apelin receptor agonist—BMS-986224—as a potential target in HF. Its administration enhances cardiac output by different mechanisms compared to ACE-I. BMS-986224 is a selective and potent apelin agonist, with a receptor binding profile similar to that of apelin-13. In experimental models, BMS-986224 perfusion increased stroke volume and cardiac output but, unlike ACE-I, it did not prevent cardiac hypertrophy and fibrosis. The molecule is presented as a new, potent non-peptide apelin agonist, with oral bioavailability that mimics the signaling properties of apelin-13. Its oral administration induces a sustained increase in cardiac output, the unique profile supporting its further clinical evaluation in HF [34].

Omecamtiv mecarbil, a selective cardiac myosin activator that improves cardiac contractility, is another promising molecule in HFrEF. The GALACTIC-HF (Global Approach to Lowering Adverse Cardiac Outcomes Through Improving Contractility in Heart Failure), a randomized, placebo-controlled, phase 3 trial, showed a lower risk of heart-failure events and cardiovascular death with omecamtiv mecarbil compared to placebo. The trial enrolled 8256 patients with NYHA class II, III or IV symptoms, LVEF 35% or less and elevated natriuretic peptides who were then assigned to a dose of 25 mg, 37.5 mg or 50 mg twice daily or to a placebo dose. The median follow-up time was 21.8 months. The primary composite endpoint was an HF event or cardiovascular death which occurred in 37.0% of the omecamtiv mecarbil group versus 39.1% in the placebo group (hazard ratio, 0.92; 95% CI, 0.86 to 0.99; *p* = 0.03). Omecamtiv mecarbil produced greater therapeutic benefit and was more efficient in patients with an LVEF of 28% or less (hazard ratio, 0.84; 95% CI, 0.77–0.92) and blood pressure lower than 100 mmHg (hazard ratio, 0.81; 95% CI, 0.70–0.94), with no effect on cardiovascular death rates. In addition, omecamtiv mecarbil had no adverse effect on blood pressure, heart rate or creatinine and potassium levels. The drug is still awaiting FDA approval [51].

Cell and gene therapy is making amazing progress in experimental research, but currently in clinical trials the results remain modest. The theoretical premise is that, in humans, the cardiomyocytes lost after myocardial infarction are replaced only by fibrous scars and hypertrophy of remaining cells, while inferior species have a powerful regenerative capacity. Some phase 1, 2 and 3 studies are testing cell therapy (progenitor cells, uni- and multipotent cardiomyocytes, tissues processed by cell engineering techniques), gene therapy (noncoding RNA) or noncellular therapy such as growth factors. While current pharmacotherapy manages to prolong life and avoids unwanted major events without addressing the cause of HF—loss of myocardial contractility—cell and gene therapy mainly address this cause while aiming for the restoration of lost cardiomyocytes [52,53].

The loss of viable cardiomyocytes is a central feature of cardiac remodeling and progression to HF that occurs secondary to the process of necrosis or apoptosis. The triggers are not fully understood but several mechanisms have been suggested, including the gradual accumulation of oxidative stress-related damaged macromolecules, persistent hyperactivation of catecholamines, activation of TNF-α signaling and chronic inflammatory signaling [54]. Mitochondrial permeability transition pores (MPTPs) play a critical role in cell death, and several methods of inhibiting this process (e.g., inhibition of CaMKII31 or cyclophilin D) have been shown to prevent cell death or reduce adverse remodeling in experimental studies. Dysfunctional mitochondria resulting from the intracellular damage caused by oxidative stress are also considered to be a central player in inducing the process called sterile inflammation. Incomplete mitochondrial DNA autophagy can activate the TLR 9 receptor (Toll-like receptor), a signaling cascade that leads to the production of proinflammatory cytokines and continuous cell damage in HF. Similarly, activation of inflammasomes, multiprotein complexes that identify harmful substances resulting intracellularly through oxidative stress, are also involved in ischemic HF [55]. Therefore, targeting inflammatory receptors and cytokine signaling may provide potential resources to limit cell death in HF. Autophagy is a key process in cell renewal. Both defective and excessive autophagy have a negative impact on cardiac remodeling and progression to HF. Rapamycin complex (mTOR) is the key regulator of autophagy signaling, currently considered a potential therapeutic target. Cell viability is also determined by other cell processes, such as the ubiquitin-proteasome system (UPS) and the endoplasmic reticulum (ER) stress response, both involved in maintaining intracellular protein homeostasis. Disruption of these mechanisms also leads to the activation of cell death pathways. Modulation of UPS and ER signaling cascades, as well as their associated molecules, may be another potential target for HF therapy [56].

## 6. Conclusions

Regarding the development of HF strategies, what Moses Maimonides said over 800 years ago in his oath is still valid: “Grant me the strength, time, and opportunity always to correct what I have acquired, always to extend its domain; for knowledge is immense and the spirit of man can extend indefinitely to enrich itself daily with new requirements. Today he can discover his errors of yesterday and tomorrow he can obtain a new light on what he thinks himself sure of today” [57].

HF management underwent dynamic changes regarding therapeutic concepts, from the hemodynamic and neurohormonal treatment to strategies targeting maladaptive signaling pathways. Recent beneficial results with ARNI and SGLT2 inhibitors support the fact that improving HF progression is not an impossible task and significant progress is going to be made. The current paradigm is based on creating a balance between the classic hemodynamic and neurohormonal modulators and the new molecular targets in order to obtain a personalized, adapted therapy. Three different conceptual therapeutic models (hemodynamic, cardiorenal and neurohormonal models) were used to develop strategies for treating HFrEF, but none of these can explain disease progression completely. New therapeutic classes (ARNI, Vericiguat, Omecamtiv mecarbil) were also developed based on interesting observations provided by current models, whereas the pleiotropic mechanisms underlying the benefits of SGLT2 inhibitors are not fully understood. Thus, it is an urgent need to rethink or expand the existing models or develop new paradigms for additional effective and safe therapies for this population [58]. Given its complex pathophysiology, the fundamental challenge is to adapt the medication to patient phenotype. For clinicians, it is a difficult task that requires an integrative but personalized view. On the other hand, the development of valid targets for HF therapy needs appropriate and complete preclinical studies based on evolving guidelines recommendations [59].

The ultimate purpose is to facilitate the prevention and progression of HF by translating research into practice. Many innovative programs have been developed to improve clinician adherence to evidence-based clinical guidelines. The National Heart, Lung, and Blood Institute emphasizes the urgent need for the development of comprehensive guidelines reflecting real-life clinical scenarios to assist physicians in improving daily practice [60].

This review summarizes the molecules recently introduced in clinical practice guidelines for HFrEF, but also the potential of old and new targets. The purpose is to provide updated information and advocate for its implementation in clinical practice for optimal care of patients with heart failure.

## Figures and Tables

**Table 1:** Pathophysiological mechanisms in HF.

Classic Pathophysiological Changes	Modern Pathophysiological Mechanisms
Hemodynamic changes Neurohumoral activation: - sympathetic nervous system - renin-angiotensin-aldosterone system - vasopressin - endothelin system - natriuretic peptide system - nitric oxide, prostaglandins, bradykinin	Inflammation: cytokine release (TNF-α, interleukins) Release of oxygen free radicals Endothelial dysfunction - vasoconstriction, remodeling (hypertrophy, myocyte apoptosis, extracellular matrix remodeling) - alteration of cGMP activity, protein kinase G versus protein kinase C - activation of endothelins - release of metalloproteinases Myocardial energy dysfunction - alteration of mitochondrial oxidative metabolism - reduction in fatty acid oxidation - decrease in ATP deposits - SERCA2a deficiency and poor calcium handling - disorders of excitation–contraction coupling Alterations in gene expression and cellular signaling

**Table 2:** Major clinical trials with guideline-recommended drugs (except ACE-I/ARB, beta blockers and spironolactone) in HFrEF.

Trial	EPLERENONE	IVABRADINE	EPLERENONE	ARNI	SGLT2i					VERICIGUAT
	EPHESUS	SHIFT	EMPHASIS-HF	PARADIGM-HF	EMPA-REG Outcome	CANVAS Program	DECLARE TIMI 58	DAPA-HF	EMPEROR-Reduced	VICTORIA
Year	2003	2010	2011	2014	2015	2017	2018	2019	2020	2020
Number of patients	3313	6558	2737	8442	7020	10142	17160	2373	3730	5050
Treatment regimen	Eplerenone vs. placebo	Ivabradine vs. placebo	Eplerenone/ placebo	Sacubitril/ valsartan vs. enalapril	Empagliflozin vs. standard treatment	Canagliflozin vs. standard treatment	Dapagliflozin vs. standard treatment	Dapagliflozin vs. standard treatment	Empa gliflozin vs. placebo	Vericiguat /placebo
Follow-up time	16 months	22.9 months	21 months	27 months	37.2 months	28.8 months	74.4 months	18.2 months	16 months	10.3 months
Inclusion criteria	NYHA class III, IV LVEF < 35%, 3–14 days after acute myo-cardial infarction	NYHA class II-IV, LVEF < 35% Sinus rhythm Heart rate ≥ 70/per minute, hospitalization for HF within the previous year, on stable back-ground treatment including a beta blocker if tolerated	NYHA class II, history of chronic systolic HF of at least 4 weeks duration Ischemic/ non-ischemic etiology Optimal dose /maximally tolerated dose of standard HF therapy	NYHA class II-IV symptoms, LVEF ≤ 40% until 2010 and ≤35% after If no HF hospitalizations in prior year: BNP ≥ 150 pg/mL or NT-proBNP ≥ 600 pg/mL If a HF hospitalization in prior year: BNP ≥ 100 pg/mL or NT-proBNP ≥ 400 pg/mL ACE-I/ARB, beta blocker therapy	DM 2 HbA1c of ≥7.0% background glucose-lowering therapy unchanged for ≥12 weeks prior to randomization or, in the case of insulin, unchanged by >10% from the dose at randomization in the previous 12 weeks Body mass index ≤45 kg/m^2^ eGFR > 30 mL/min/1.73m^2^ Established CV disease	HbA1c ≥ 7.0% to ≤10.5% eGFR ≥ 30 mL/min/1.73 m^2^ Age ≥ 30 years and history of prior CV event or age ≥ 50 years with ≥2 CV risk factors	Age ≥ 40 years, DM2 HbA1c of ≥6.5% and ≤12%, eGFR of >60 mL/min /1.73 m^2^ Established CV disease/multiple risk factors including men ≥ 55 years or women ≥ 60 years with HT, dyslipidemia or tobacco use	LVEF ≤ 40% NT-proBNP ≥ 600 pg/mL or ≥900 pg/mL if atrial fibrillation	NYHA class II, III LVEF ≤ 40% Elevated NT-proBNP eGFR > 20 mL/min/1.73 m^2^ Guideline recommended medical therapy stable 1 week prior to first visit	Chronic HF, NYHA class II-IV LVEF < 45% and guideline-directed HF therapy Recent HF hospitalization or intravenous diuretic use Elevated natriuretic peptides
Exclusion criteria	Use of potassium-sparing diuretics A serum creatinine concentration > 2.5 mg/dL A serum potassium concentration > 5.0 mmol/L	Recent myocardial infarction Ventricular/ atrioventricular pacing that is operative for more than 40% of the day, atrial fibrillation hypotension	Severe chronic systolic HF symptomatic at rest despite optimal medical therapy eGFR < 30 mL/min/1.73 m^2^	Symptomatic hypotension SBP < 100 mmHg at screening or <95 mmHg at randomization eGFR < 30 mL/min/1.73 m^2^ Reduction in eGFR > 25% serum potassium level >5.2 mmol/L History of angioedema Unacceptable side effects with ACE-I or ARB	Uncontrolled hyperglycemia, liver disease Planned cardiac surgery or angioplasty within 3 months, bariatric surgery within the past 2 years and other gastrointestinal surgeries that induce chronic malabsorption. Cancer treatment with anti-obesity drugs, alcohol/drug abuse within the last 3 months Acute coronary syndrome, stroke/ transient ischemic attack within 2 months prior to informed consent	History of diabetic ketoacidosis DM 1 Pancreas or beta cell transplantation, or diabetes secondary to pancreatitis or pancreatectomy, severe hypo-glycemic episode within 6 months before screening	DM 1 Bladder cancer Radiation therapy to the lower abdomen or pelvis at any time Chronic cystitis and/or recurrent urinary tract infections, pregnant or breast-feeding patients	eGFR < 30 mL/min/ 1.73 m^2^ and SBP < 95 mmHg	Myocardial infarction Coronary artery bypass graft surgery, stroke Heart trans-plantation Acute decompensated HF SBP ≥ 180 mm Hg at visit 2 Symptomatic hypotension and/or a SBP < 100 mmHg, liver disease Impaired renal function defined as eGFR < 20 mL/min/1.73 m^2^ Use/prior use of a SGLT2i, pregnancy	Use of long-acting nitrates, phosphodiesterase type 5 inhibitor, riociguat Heart transplantation Continuous intravenous diuretics eGRF 15 mL/min/1.73 m^2^ or dialysis Severe pulmonary disease requiring continuous oxygen Severe hepatic insufficiency
Primary endpoint	Death from any cause and death from CV causes or HF hospitalization	CV death or hospital admission for worsening HF	Death from CV causes or hospitalization for HF	CV mortality or HF hospitalization	MACEAll-cause mortality or CV mortality, myocardial infarction, stroke CV hospitalization Disease Progression or renal mortality	MACE All-cause mortality/ CV mortality Myocardial infarction, stroke CV hospitalization Disease progression or renal mortality	MACEAll-cause mortality/ CV mortality. Myocardial infarction Stroke CV hospitalization Disease progression or renal mortality	Hospitalizationor visit to the emergency room due to HF Hospitalization for HF Visit to the emergency room due to HF or CV death	CV death or hospitalization for worsening HF	CV death or HF hospitalization
*p* (superiority)	RR 0.87; 95 CI 0.79 to 0.95; *p* = 0.002	HR 0.82, 95% CI 0.75–0.90, *p* < 0.0001	HR 0.63; 95% CI 0.54 to 0.74; *p* < 0.001	HR 0.80; 95% CI 0.73 to 0.87; *p* < 0.001	HR 0.86; 95.02% CI, 0.74 to 0.99; *p* = 0.04 for superiority	HR 0.78; 95% CI 0.67–0.91, *p* = 0,02	CI < 1.3; *p* < 0.01 for non-inferiority	HR 0.74; 95% CI 0.65 to 0.85; *p* < 0.01	HR 0.75; 95% CI 0.65 to 0.86; *p* < 0.001	HR 0.90; 95% CI 0.82 to 0.98; *p* = 0.02
Secondary endpoint	Death from any cause or any hospitalization	Hospital admissions for worsening HF/ deaths due to HF	All-cause mortality or HF hospitalization	CV mortality HF hospitalization All-cause mortality	Hospitalization due to HF				Total hospitalizations Composite renal outcome	Death from CV causes Hospitalization for HF
*p*	RR 0.92; 95 CI 0.86 to 0.98; *p* = 0.02	21% placebo vs. 16% with ivabradine; HR 0.74, 0.66-0.83; *p* < 0.0001	19.8% vs. 27.4%, HR 0.65; 95% CI 0.55 to 0.76, *p* < 0.001	HR for death from any cause, 0.84; 95% CI, 0.76 to 0.93; *p* < 0.001	2.7% and 4.1%, respectively; 35% RR reduction *p* = 0.08	HR, 0.70; 95% CI, 0.55–0.89, *p* < 0.001	4.9% vs. 5.8%; HR, 0.83; 95% CI, 0.73 to 0.95; *p* = 0.005	-	HR 0.70; 95% CI, 0.58 to 0.85; *p* < 0.001	HR 0.90; 95% CI, 0.83 to 0.98; *p* = 0.02

ACE-I—angiotensin converting enzyme inhibitors, ARB—angiotensin receptor blockers, BNP—brain natriuretic peptide, CI—confidence interval, CV—cardiovascular, DM 2—diabetes mellitus type 2, eGFR—estimated glomerular filtration rate, HbA1c—glycated hemoglobin C, HF—heart failure, HFrEF—heart failure with reduced ejection fraction, HR—hazard ratio, HT—hypertension, LVEF—left ventricular ejection fraction, MACE—major adverse cardiac events, NYHA—New York Heart Association, RR—relative risk, SBP—systolic blood pressure, SGLT2i—sodium-glucose cotransporter 2 inhibitors.

**Table 3:** Clinical trials targeting old drugs, new drugs and potential targets in HFrEF.

	Objective/Results	Number of Patients	Year of Completion
NCT03783429 (Digoxin Evaluation in Chronic Heart Failure: Investigational Study In Outpatients in the Netherlands (DECISION) https://clinicaltrials.gov/ct2/show/NCT03783429 (accessed on 3 July 2022)	To evaluate whether lower doses of digoxin, guided by serum concentrations, will reduce HF hospitalizations and cardiovascular death rate.	recruiting	2025
TRANSFORM-HF (ToRsemide compArisoN With furoSemide FORManagement of Heart Failure) [27] https://clinicaltrials.gov/ct2/show/NCT03296813 (accessed on 3 July 2022)	To compare Torsemide efficiency to Furosemide and its effects on mortality and morbidity.	2859	2022
ADVOR (Acetazolamide in Decompensated Heart Failure With Volume OveRload) [28] https://clinicaltrials.gov/ct2/show/NCT03505788 (accessed on 3 July 2022)	To test the efficiency of the association of Acetazolamide in HF.	519	2022
QUEST (Efficacy and Safety of Tolvaptan in Heart Failure Patients with Volume Overload Despite the Standard Treatment with Conventional Diuretics) [29] https://clinicaltrials.gov/ct2/show/NCT01651156 (accessed on 3 July 2022)	To evaluate the efficacy and safety of tolvaptan in HFrEF patients with cardiac edema after current diuretic treatment. Increase in total kidney volume: 2.8% per year (95% CI, 2.5 to 3.1) in the tolvaptan group vs. 5.5% per year in the placebo group (95% CI, 5.1 to 6.0; *p* < 0.001). The composite endpoint favored tolvaptan over placebo (44 vs. 50 events per 100 person-years, *p* = 0.01), with lower rates of worsening kidney function (2 vs. 5 events per 100 person-years, *p* < 0.001) and kidney pain (5 vs. 7 events per 100 person-years, *p* = 0.007).	244	2013
EVEREST (The Efficacy of Vasopressin Antagonism in Heart Failure Outcome Study With Tolvaptan) [29] https://clinicaltrials.gov/ct2/show/NCT00071331 (accessed on 3 July 2022)	To compare the safety and efficacy of tolvaptan versus placebo in the treatment of patients with worsening congestive HF. The composite endpoint of cardiovascular death or hospitalization for HF: 871 tolvaptan group patients (42%) and 829 placebo group patients (40.2%) (hazard ratio, 1.04; 95% CI, 0.95–1.14; *p* = 0.55).	3600	2006
NCT03797001—Interleukin-1 Blockade in Recently Decompensated Heart Failure-2 (REDHART2) [30] https://clinicaltrials.gov/ct2/show/NCT03797001 (accessed on 3 July 2022)	To evaluate the effects of anakinra (100 mg subcutaneous injection, daily for 24 weeks) on peak aerobic exercise capacity measured with a cardiopulmonary test after 24 weeks in patients with recently decompensated HFrEF and increased systemic inflammation.	102	2024
FAIR-HF2 (Intravenous Iron in Patients With Systolic Heart Failure and Iron Deficiency to Improve Morbidity and Mortality) https://clinicaltrials.gov/ct2/show/NCT03036462 (accessed on 3 July 2022)	To investigate the effect of a long-term therapy with ferric carboxymaltosis vs. placebo on decreasing the rate of recurrent hospitalizations and CV death in HfrEF.	recruiting	2024
NCT03388593 (Survival Study of the Recombinant Human Neuregulin-1β in Subjects With Chronic Heart Failure) https://clinicaltrials.gov/ct2/show/NCT03388593 (accessed on 3 July 2022)	To test whether daily intravenous neuroregulin 1 perfusion, followed by weekly bolus, is feasible and safe in HFrEF.	1600	2023
NCT03875183-Study to Evaluate Effects of INL1 in Patients With Heart Failure and Reduced Ejection Fraction (TRACER-HF) [31] https://clinicaltrials.gov/ct2/show/NCT03875183 (accessed on 3 July 2022)	To evaluate the efficacy and safety of three PO INL1 doses in HFrEF. The primary outcome measure is NT-proBNP serum level decrease. The secondary outcome measures are echocardiographic parameters and functional status changes.	200	2023
HEART-FID (Randomized Placebo-controlled Trial of FCM as Treatment for Heart Failure With Iron Deficiency) https://clinicaltrials.gov/ct2/show/NCT03037931 (accessed on 16 July 2022)	To evaluate the effects of intravenous ferric carboxymaltose FCM vs. placebo on the 12-month rate of death, hospitalization for worsening HF and the 6MWT distance in HfrEF patients with iron deficiency.	active, not recruiting 3068 participants	2023
IRONMAN (Intravenous Iron Treatment in Patients With Heart Failure and Iron Deficiency) https://clinicaltrials.gov/ct2/show/NCT02642562 (accessed on 3 July 2022)	To evaluate the additional effect of intravenous iron (ferric derisomaltose) vs. placebo on top of standard care in HFrEF patients with iron deficiency.	active, not recruiting 1160 participants	2022
NCT03888066—DIAMOND (Patiromer for the Management of Hyperkalemia in Subjects Receiving RAASi Medications for the Treatment of Heart Failure) [32] https://www.clinicaltrials.gov/ct2/show/NCT03888066 (accessed on 3 July 2022)	To evaluate patiromer compared to control among patients with HFrEF and a history of hyperkalemia. The primary endpoint, adjusted mean change in serum potassium level, was 0.03 mEq/L in the patiromer group vs. 0.13 mEq/L in the control group (*p* < 0.001).	878	2022
ARTS-HF (MinerAlocorticoid Receptor antagonist Tolerability Study-Heart Failure) [33] https://clinicaltrials.gov/ct2/show/NCT04435626 (accessed on 3 July 2022)	To investigate the safety and potential efficacy of finerenone in patients with worsening chronic HFrEF and at high risk of hyperkalaemia and worsening renal dysfunction. Finerenone demonstrated a decrease of >30% in plasma N-terminal pro-B-type natriuretic peptide during 90 days in 37.2% of patients vs. eplerenone. The composite endpoint (CV hospitalizations, acute worsening HF or 90-day all-cause mortality) was statistically significant only in the 10 to 20 mg group (hazard ratio 0.56, 95% CI, 0.35; 0.90; *p* = 0.02).	1066	2021
GALACTIC-HF (Global Approach to Lowering Adverse Cardiac Outcomes Through Improving Contractility in Heart Failure) [34] https://clinicaltrials.gov/ct2/show/NCT02929329 (accessed on 3 July 2022)	To evaluate the selective cardiac myosin activator omecamtiv mecarbil compared to placebo among patients with HFrEF. The primary composite endpoint: omecamtiv mecarbil reduced CV death or HF events compared to placebo (hazard ratio 0.92 [95% CI, 0.86–0.99]; *p* = 0.02).	8256	2021
CANTOS (Cardiovascular Risk Reduction Study (Reduction in Recurrent Major CV Disease Events) [35] https://clinicaltrials.gov/ct2/show/NCT01327846 (accessed on 3 July 2022)	To test if canakinumab would prevent hospitalization for HF and the composite of HHF or HF-related mortality. A dose of 150 mg every 3 months significantly reduced the composite endpoint of HF hospitalization or HF–related mortality in patients with a history of acute myocardial infarction (hazard ratio vs. placebo, 0.83; 95% CI, 0.73 to 0.95; *p* = 0.005).	10,061	2020
ISRCTN94506234 (Q-SYMBIO trial) https://www.isrctn.com/ISRCTN94506234 (accessed on 3 July 2022)	To evaluate coenzyme Q10 as an adjunctive treatment in chronic HFrEF. Improvement of composite risk assessed by MACE (HR: 0.23; 95% CI = 0.11–0.51, *p* < 0.001). Improvement in NHYA class after 2 years of CoQ10 supplementation vs. placebo (48% vs. 25%, *p* = 0.003). Significant improvement in LVEF in Coq10 group of 6% from baseline (*p* = 0.021).	420	2019
REDHART (REcently Decompensated Heart failure Anakinra Response Trial) [36] https://clinicaltrials.gov/ct2/show/NCT01936909 (accessed on 3 July 2022)	To test inhibition of inflammatory response and improvement in peak aerobic exercise capacity in recently decompensated HFrEF after administration of IL-1 receptor antagonist (anakinra). Anakinra improved peak aerobic exercise capacity after 12 weeks of treatment in patients with LVEF < 50% (from 14.5 mL/kg/minute to 16.1 mL/kg/minute; *p* = 0.009).	60	2017
CONFIRM-HF (Ferric CarboxymaltOse evaluatioN on perFormance in patients with IRon deficiency in coMbination with chronic Heart Failure) [37] https://clinicaltrials.gov/ct2/show/NCT01453608 (accessed on 16 July 2022)	To determine, relative to placebo, the effect of intravenous ferric carboxymaltose (FCM) over a 1 year period on exercise capacity in patients with chronic heart failure and iron deficiency. FCM significantly prolonged 6MWT distance (difference FCM vs. placebo 36 ± 11 m, *p* < 0.001) A reduction in the risk of hospitalizations for worsening HF (hazard ratio-95% confidence interval: 0.39 (0.19–0.82), *p* = 0.009); a significant improvement in NYHA class symptoms and quality of life scores.	304	2015
NCT00454818—Efficacy and Safety Study of Genetically Targeted Enzyme Replacement Therapy for Advanced Heart Failure (CUPID) [34] https://clinicaltrials.gov/ct2/show/NCT00454818 (accessed on 3 July 2022)	To evaluate the effects of 3 doses of AAV1/SERCA2a versus placebo in patients with HF NYHA class III, IV, LVEF ≤ 35%, $\dot{V}$o2max ≤ 20 mL/kg per minute and ICD on optimal therapy. Primary endpoints: incidence of treatment adverse events at 12 months; length of CV-related hospitalizations at 6 months; change in NYHA class, MLWHFQ score, 6-min walk test, $\dot{V}$o2max, absolute levels of NT-proBNP, LVEF, LVESV at 6 months. Results: significant decrease in clinical events, hospitalization length and NT-proBNP levels, trending toward significant recovery of clinical evolution and functional capacity.	51	2012
NCT00841139 (Metabolic Manipulation in Chronic Heart Failure) [38] https://clinicaltrials.gov/ct2/show/NCT00841139 (accessed on 3 July 2022)	To test whether short-term treatment with perhexiline improves cardiac energetics, LVEF or symptoms of HF by altering substrate utilization. Perhexiline improves cardiac energetics (30% increase in the phosphocreatine/adenosine triphosphate ratio from 1.16 ± 0.39 to 1.51 ± 0.51; *p* < 0.001) and symptom status (*p* = 0.036) with no evidence of altered cardiac substrate utilization or changes in LVEF.	50	2011
FAIR-HF (Ferinject^®^ Assessment in Patients With Iron Deficiency and Chronic Heart Failure) [31] https://clinicaltrials.gov/ct2/show/NCT00520780 (accessed on 16 July 2022)	To evaluate the efficacy of Ferinject^®^ in improving symptoms of chronic HFrEF in patients with iron deficiency. Results: significant improvements in NYHA functional class at week 24 (odds ratio for improvement by one class, 2.40; 95% CI, 1.55 to 3.71; *p* < 0.001), in distance on the 6MWT and in quality of life at week 24 (*p* < 0.001 for all comparisons).	456	2009

CV—cardiovascular, HF—heart failure, HFrEF—HF with reduced ejection fraction, ICD—implantable cardiac defibrillators, IL—interleukin 1, LVEF—left ventricular ejection fraction, LVESV—left ventricular end systolic volume, MACE—major adverse cardiovascular events, MLWHFQ—Minnesota Living With Heart Failure Questionnaire, MRA—mineralocorticoid receptor antagonist, RAASi—renin angiotensin aldosterone inhibitors, $\dot{V}$o2max—maximal oxygen uptake.

## Data Availability

Not applicable.
